# Overview on Innovative Packaging Methods Aimed to Increase the Shelf-Life of Cook-Chill Foods

**DOI:** 10.3390/foods10092086

**Published:** 2021-09-03

**Authors:** Maria Lisa Clodoveo, Marilena Muraglia, Vincenzo Fino, Francesca Curci, Giuseppe Fracchiolla, Filomena Faustina Rina Corbo

**Affiliations:** 1 Interdisciplinary Department of Medicine, University Aldo Moro Bari, 70125 Bari, Italy; marialisa.clodoveo@uniba.it; 2 Department of Pharmacy-Drug Sciences, University Aldo Moro Bari, 70125 Bari, Italy; vincenzo.fino@gmail.com (V.F.); francesca.curci@uniba.it (F.C.); giuseppe.fracchiolla@uniba.it (G.F.); filomena.corbo@uniba.it (F.F.R.C.)

**Keywords:** cook–chill technology, foods, packaging, shelf-life, sustainability

## Abstract

The consumption of meals prepared, packaged, and consumed inside and outside the home is increasing globally. This is a result of rapid changes in lifestyles as well as innovations in advanced food technologies that have enabled the food industry to produce more sustainable and healthy fresh packaged convenience foods. This paper presents an overview of the technologies and compatible packaging systems that are designed to increase the shelf-life of foods prepared by cook–chill technologies. The concept of shelf-life is discussed and techniques to increase the shelf life of products are presented including active packaging strategies.

## 1. Introduction

### 1.1. Food Away from Home (FAFH) vs. Food Packaging Technology

The consumption of food away from home (FAFH) is constantly rising in the whole industrialized society because of the increasing need to carry out activities for both children at school and adults at work [1,2]. A rational approach should take in account innovative methods for cooking food stuff and new packaging. Foods are expected to be tasty and healthy even if not cooked at the moment of consumption. A rational design for cooking and store foods could reduce waste and containing food expenditure. Food packaging technology research should help food manufacturers to reach this goal meeting the needs of consumers, the society and the manufacturers, often very different from each other.

While the consumers are oriented to products that match their own lifestyle, the society needs safer and environmentally sustainable products that means major attention also on packaging material, not only on food stuff. On the other hand, the manufacturers are interested in the use of better and more cost-effective packaging materials and technologies to meet the market demand and make profits [3,4].

### 1.2. Food, Energy, Environment Trilemma

In the next decades, the global community will attend to an increasing of food demand, due to the third world demographic explosion, and the displacement of food crops, due to a change of the land use that will be directed to the biofuel and bioenergy production [5]. A trend is visible in the scientific literature showing the conflict between food production and bioenergy/biofuel production is the so-called “*food, energy, environment trilemma*” [6,7]. One of the possible strategies that should be used to solve this problem is the enhancement of the food transformation efficiency intended as a reduction of waste and, therefore, a general reduction of losses in edible material during food manipulation and consumption.

Now larger and medium sized catering and hospitality companies are forced to use new technologies for cooking and storage due to business needs. Maybe in the near future, small companies, restaurants, and small communities including families will embrace this new “food preparation philosophy” in order to reduce food costs.

The cook–chill processing procedure has been widely used in catering since the early Nineties [8] coming from the practice of serving unused food the day after preparation to avoid waste. This procedure consists in rapid cooling of cooked food to 2–4 °C before or after packaging that will be stored at the same temperature until it will be served both hot (by warming at 70 °C) or cold.

The current paper presents an overview of the advances in the cook–chill technology developed to increase the shelf-life of foods without loss of their nutritional and organoleptic value.

## 2. The Cook–Chill Technology

The habit of consuming food is a common practice for households who have access to refrigeration. At the same time, lack of adequate preparation and storage can result in incidents of food poisoning [9]. This can also result in considerable food spoilage which contributes to increasing food wastage.

**Figure 1:**
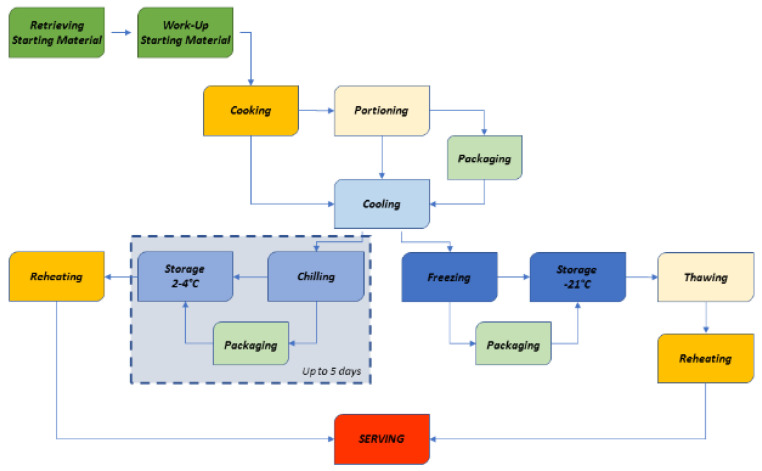
A block scheme of the principal steps in cook–chill procedure.

Companies involved in serving meals have implemented specific technologies of the last two or three decades able to prepare and package meals designed for reheating without loss of taste, flavor and texture. To obtain this result it is strictly necessary to follow a simple protocol that is schematically shown in Figure 1.

### 2.1. Retrieving and Work-Up of Raw Materials

Choice, storage, handling, and preparation operations of raw materials follow simple hygienic rules principally derived from good sense and coded by administration such as U.S. Food and Drug Administration (FDA) or European Food Safety Authority (EFSA) (see legislation paragraph below). The standard quality of starting materials must be as high as possible because it is not going to improve with cooking, so the systematic control on the supplier chain guarantees to keep the quality level.

After selection of raw materials, it is necessary to follow the basic food safety principles, to ensure that the proper temperature and humidity of the starting materials are kept. In addition, in preparing procedures, basic food safety principles have to be applied: separate work surfaces and dedicated utensils should be used for different types of food (fish, meat, poultry and vegetables) to prevent cross-contamination. At the best conditions, food preparation should take place in a separate area from cooking and post-cooking.

Frozen raw materials should be completely thawed out before use, rapid high temperature thawing should be avoided because it can allow the growth of pathogens [10,11,12] and may leave cold spots at the core of the food. Thawing with microwave ovens is generally not recommended for the same reasons [7,13,14].

### 2.2. Cooking

Almost all kinds of cooking procedures are compatible with the cook–chill procedure, this fact makes it a very flexible tool for food manufacturers. The main and unique problem that it must be faced is the temperature control at the core of the food to ensure the proper destruction of pathogens that may be present inside it [15].

The control of temperature depends on the type of food, its thickness, the type of pathogens that eventually present, its charge, and cooking methods [16,17,18,19,20,21,22,23].

### 2.3. Cooling

The cooling step of the procedure is the most important of the entire process and it can be carried out both before or after portioning and/or packaging of the cooked food.

The chilling apparatus that must be used in a cook–chill procedure should be capable of chilling the food to around 3 °C in 2–4 h in dependence of the just cooked food temperature. The rapid chill of the food avoids bacterial growth and preserves the appearance, texture, flavor and nutritional value of the food [24].

Food probes are frequently used to check the process, because there are plenty of variables that should influence the chill rate. If we only consider the type of food and the cooking procedure followed in different recipes, we should have a too large volume of information that we have to manage. Furthermore, each kind of food presents different chill problems related principally on the nutrient content, on the water amount and its quantity. Liquid preparation can be efficiently cooled by adding microbiologically pure slush ice to concentrated soup dishes, while solids foods such as meat, fish or vegetables are conveniently air-chilled [18].

Heat conduction by air circulation depends on surface area for heat exchange, gradient temperature between hot surfaces and air flow, surface heat transfer coefficient.

Each product-refrigerating system couple can be characterized by an own surface heat transfer coefficient that depends on the velocity impressed to the air flow and on its specific heat. This constant value varies in a range from 5 to 500 W·m^−2^·K^−1^ [25].

With the aim to improve the performance of the chilling technology a lot of computational studies were performed on fluid dynamics [26,27,28,29,30] that presumably will influence the next generation chilling technology.

While the improvement of the shelf-life of food using a cold chain after the cooking process is a simple idea, the industrial or semi-industrial scale-up process is not so easy. The rapid and homogeneous reduction of food temperature appear to be the main problem of the entire process involving a lot of factors ascribed to the intrinsic nature of the foodstuff and its variability, so the equipment should be able to be used to apply the procedure to the highest possible number of recipes to make the process economically favorable for foodservice industries.

## 3. Patent in Cook–Chill Technology

The following sections present a brief overview of the recent patent activity in food service and catering. Patents related to both equipment designed for the cook–chill process and other types of facilities useful for heat removal, cooking, sterilization or storage of low temperatures that could be used in the same process are discussed.

### 3.1. Cook–Chill Apparatus

This group of inventions includes both cooking apparatus for low scale food production than very large devices useful for industrial purposes. An example of a low scale apparatus is a device consisting in a plate with a lower multizonal heater that can cooks two different recipes at the same time divided in two different vessels. It has also an upper heater for grilling [31,32,33].

Several inventions are based on the same principle, this kind of apparatus consists in a water bath (typically a tank) in which packaged food can be cooked and rapidly chilled changing the temperature of the bath. Inventions differ between them in the warming system [34], water filling and replacement devices, and temperature control devices [35,36,37,38]. Some similar apparatus was designed also for unpackaged food [39].

### 3.2. Cooker/Rethermalizer Systems

Cooker or re-thermalizer devices were designed in a similar way in respect to the cooker–chiller devices for packaged food. Food fills racks in heating liquids filled tanks. The bath temperature is controlled by temperature sensor devices [40,41,42,43,44,45].

### 3.3. Storage

Cook–chill technologies rely on the containment of foods and would not be possible without materials that can withstand cooking and storage temperatures.

These items can be tubular bags with one end open and the other sealed with a heat seal. The bags consist of a multilayered co-extruded plastic film made of various inert polymers such as nylon, polyethylene, polyesters used alone or in various combinations between them [46,47,48,49].

### 3.4. Procedures

This section presents some examples of complementary inventions (devices and protocols) in work-up procedures of cook–chill processes.

One of these is a device for distributing cooled food, for large kitchens, comprising a belt conveyor, several mobile feed distributor devices and a refrigerator circuit closed with a refrigeration system. The cooling capacity of the system can be divided into several cooling points which are arranged in the longitudinal direction of the belt conveyor and have means for connecting a feed distributor apparatus [50]. A computer program for management of out-of-hospital cooking service that can on-line receive meal orders from a plurality hospital is described in a patent work [51]. This system can efficiently send information to large scale kitchens that cook and deliver daily meal to each patient that is registered on a master file containing nutritional and eat data according to medical prescriptions.

In addition, small companies such as restaurants can follow industrial protocol for preparing pre-cooked food [52] dividing the process in sequential steps: food cooking, portioning and packaging, removing oxygen by vacuum pump, pasteurization or sterilization and blast-chilling of the packages.

## 4. The Shelf-Life of Foods

### 4.1. Shelf-Life Definition

A unique definition of shelf-life does not exist. Generally, it could be defined as the amount of time in which a useable wholesome state under the expected conditions of storage using agreed upon methods and acceptance criteria [53,54].

In the last 25 years, experts and institutions have provided several definitions speaking in term of acceptable sensory and nutritional properties of foods, or general eligibility under defined environmental conditions.

Consumer preferences and lifestyles have impacted food product formulation, preparation, and consumption habits, and this too provides the impetus for renewed focus on shelf-life determination. Upon commercialization, shelf-life tests must be performed upon the first several lots of production in order to verify previously determined outcomes with prototypical samples. In some instances, challenge studies must be performed to validate the ongoing safety throughout shelf-life. Then for ongoing food production, routine shelf-life testing is an essential quality metric, albeit a lagging indicator of quality. Attributes measured include microbiological counts, chemical degradation, physical deterioration, and sensory properties [55].

In 1993 IFST (Institute of Food Science and Technology) for the first time identified the key factors that must be considered in the shelf-life assessing process: (a) safety; (b) sensory, chemical, physical and microbiological characteristics; and (c) nutritional label declaration.

In 2005, it was defined the sensory shelf-life [56] as the time during which the product keeps its sensory characteristics and performance declared by the manufacturer providing to the end users its benefits.

It is clear for all definitions that safety of the foods is out of discussion during this time, in other words, the food is always considered safe for a longer period than the shelf-life.

### 4.2. Law Regulation on Shelf-life

In this paragraph, we consider the law regulation of FDA and EFSA as supranational organizations belonging respectively to the United States of America and the European Union that represent a part of the world population that may influence the food trade, analyzing contact points and main differences between them.

Law regulation on labelling of information related to the shelf-life of a food product may vary quite a lot from country to country.

USDA (United States Department of Agriculture) in the United States [57] reports that product dating on food stuff is not required by federal regulation except for infant foods, so date applying on labels is a voluntary activity done by manufacturers that follow FSIS (Food Safety and Inspection Service) regulation [58].

Close to the calendar date, eventually present on the package, there must be a phrase that explains the meaning of the date shown such as, sell by, use before, or use by.

“Use by date” is an expression used mainly for infant foods that means “the last date recommended for the use of the product while at peak quality”. This is information that refers directly to the shelf-life of the product since it is not a safety date.

By the way, US-FDA (Food and Drug Administration) permits the use of this phrase on infant foods under its close control meaning that the nutritional content of the food is a safety statement for this kind of consumers and ensuring them that the formulas contain not less than the quantity of each nutrient as described on the label.

Since December 2014 [59] it was operating the regulation UE 1169/2011 in the whole EC that renewed the legislation on labelling [60] regarding all types of foods: fresh, packaged by manufactures and product by catering services.

This regulation covers all the aspects connected with product presentation and advertising. From a practical point of view [61], since the application of the regulation all food packages are deeply changed regarding its clarity (also font size character has been regulated) and completeness including the whole list of ingredients, the indication on the country of origin, nutritional facts reported as percentage of the RDA (recommended dietary allowance), the list of allergens eventually present in the food, and durability expressed in term of the date of minimum durability that indicate the date until which the food retains its specific properties when properly stored.

This kind of information is directly related to the shelf-life of the product, and it is reported on the package using the form “best before”, in which a date (with a format day-month-year) is closely reported or there is an indication on where it can be retrieved. This phrase may be substituted by “best before end” date in some specific cases. The indication on durability of highly perishable food, that represents an actual danger for human health after a short period, must be reported as “use by” date. After the “use by” date a food shall be deemed to be unsafe [62] and so it could not be considered an indication of the shelf-life of the product.

For frozen food, it is mandatory to indicate the freezing date (“frozen on” date) or the date of first freezing if the supply chain provides for more than one freezing operation.

Recently, EFSA’s Panel on Biological Hazards (BIOHAZ) provided a series of scientific opinions useful to establish guidelines on date marking and related food information in view of the implementation by food business operators (FBOs) of regulation (EU) No 1169/2011 on food information to consumers as an integral part of their food safety management system (FSMS). Specifically, the guide provides guidance on determining shelf life and storage conditions and identifying factors that affect shelf-life determination [63,64].

### 4.3. Methods to Improve Food Shelf-Life

Many different methods are known to improve the shelf-life of a food, from ancient times humans had the problem of having a safe food to eat when it was not possible to retrieve it fresh. Therefore, some of the oldest ways to treat food for storage were drying with sun exposure or by osmotic mechanisms, while in recent time cold storage, the use of chemical additives, sterilization by ionizing radiation or the use of engineered packages are normal practices for manufacturers and generally accepted by consumers.

All these methods are not suitable for all kinds of food or preparations so, it is necessary to study a way of reduction or control of the microbial charge for each case.

Food deriving from a cook–chill supply chain belongs to a special class of products for which a shelf-life enhancement would be desirable to reach as complicated to obtain. Freezing, heating or drying procedure procedures can result in significant changes to the organoleptic properties of foods [65,66]. In addition, the use of chemical additives is not very suitable because they may cause a sensory modification of the product and even if not, their presence could make the product less appealing considering today’s most prevalent consumer sensitivity [67]. European consumers, for instance, are now used to reading the product label they buy by having more awareness about the food they consume.

The use of high frequency electromagnetic radiation to sterilize cooked food is not generally applicable because the reaction that may occur on the food surface cannot be predicted [68].

The choice of the proper type of package may help to preserve cooked food, so several studies have been carried out to develop new materials and applications taking in due account the needs of consumers, the society, and the manufacturers [69,70,71]. Consumers are oriented on high quality materials that are more convenient to meet their lifestyle, the society watches on human health safety as well as friendlier products in respect to the environment to meet the needs of public and environmentalists. The manufacturers need better and more cost-effective packaging technologies to satisfy the market and make profits.

To meet the market request, developments in packaging materials have focused four main class of product that are developed in the last decades [72]:

- Sustainable packaging involves environmentally friendly technologies that are socially acceptable and economically advantageous.
- Intelligent packaging involves the use of package integrated devices such as RFID (radio frequencies identification) tags, time or temperature indicators and sensors for tracking activities or sensing the internal or external environment of the package and monitoring the product quality.
- Active packaging utilizes advanced technologies that actively modify the inner atmosphere of the package in order to extend the shelf-life of the stored product.
- Responsive food packaging in which particular materials are able to react against unfavorable stimuli in order to preserve the food quality [73].

In the following section, we will consider the field of application of packages in food technologies that are able to extend the shelf-life of cooked food and research perspectives on these engineered materials.

## 5. Food Active Packaging

The use of specific packaging could be of help in preserving the nutritional and organoleptic properties of food prepared by using cook–chill technology. In this regard, many new materials have been developed to contain food, constituting a real field of applied research that aims to introduce safe, sustainable and low-cost packaging systems to the food market. The interest in active packaging is confirmed by the increasing number of scientific papers which has more than doubled in the last decade [74,75].

Even if active functions of packaging have gained more visibility than original attributes, such as mechanical strength, barrier performance and thermal stability, every new material must satisfy each of these basic properties until it becomes a potential material for food carrying. In addition, the use and development of new packaging, as well as the use of new additives that provide interesting properties for food preservation, require careful safety and toxicological evaluations due to the potential presence of food contaminants of packaging origin on the quality and safety of fresh food [76,77,78].

To extend the shelf-life of a foodstuff it is crucial to measure the microbiological count by optimizing some factors including oxygen partial pressure, moisture and water activity, sunlight exposure and initial microbial charge [79].

Packaging with antimicrobial purposes can interact with the food contained in it or with the empty space above it, to reduce, retard or even inhibit the growth of pathogenic microorganisms and food spoilage [80].

A class of material deeply investigated for food packaging is polymers (low-density polyethylene, LDPE, in most cases) which properties such as density and permeability (related with release of small molecules) are tuned looking at the physical and chemical properties of the spread substance [81].

Polymers and composites used as emitting materials can be divided into two main categories:

- Polymers that incorporate organic compounds.
- Polymers that incorporate inorganic compounds.

Summarily, organic compounds that may be used in active packaging material, must have several features to satisfy food technology requirements and improve the shelf-life of the food itself. They must be safe for humans by ingestion, inhalation, and contact, they do not interfere with the organoleptic properties of the food, they should be able to contrast the growth of many microorganisms (bacteria and fungi) that cause food spoilage and they should have some antioxidant activity.

Synthetic additives that may be tailored with all these features have to satisfy many requirements before safety declaration (as expected for drugs). Therefore, organic compounds derived from natural sources that are deeply studied on animal models and humans, are suitable for this application.

Essential oils (EO) are mixture of compounds derived from aromatic plants that have been investigated not only for being natural product, but also because they have used since ancient time for their biological properties as antioxidant, antimicrobial, anti-tumor, analgesic, anti-pest, anti-diabetic, and anti-inflammatory [82,83,84,85].

The EOs mechanism of action has been extensively reported in the literature and concerns the breakdown of bacterial cell wall, although the effect on the destruction of enzymes or membrane proteins or the spillage of cellular content after cytoplasmic membrane breakage are also possible [83,86,87].

Our experience in evaluating EOs drug efficacy as antimicrobial agents allows us to promote their use in the engineering of new active packaging [88,89,90,91,92].

Among the class of organic additives, chitosan deserves particular attention: a polymer that shows unique characteristics and potential application.

It is directly derived from chitin, a very cheap natural occurring material which is constituted by a linear polymer of β-1,4-*N*-acetyl-D-glucosamine whose structure is close to the β-1,4-D-glucopyranoside chain of cellulose except for the acetamide group at C-2 position of the monosaccharide unit.

Chitosan and its derivatives show significant antimicrobial activity [93] alone or in combinations with essential oils [94] or other small molecules with antimicrobial properties [95,96,97]. Different polymers have been studied for food packaging, poly (butyleneadipate-co-terephtalate) (PBAT) [98,99] is used because of its mechanical properties that are comparable with LDPE. It is completely biodegradable and compostable, obtained by total metal-free processes and allowed for food application by FDA.

Cellulose is a very versatile polymer that can be obtained as a nanostructured membrane which may grow in the presence of other polymers and various additives producing bionanocomposites that may be used in food packaging technology (including active packaging) [100].

Other cellulose derivatives (hydroxypropyl-methylcellulose, HPHC, extensively used in drug formulation as cover agent for solid preparations) was recently studied [101] as matrix for controlled release of antioxidants (green tea extract) loaded on polylactic acid nanoparticles.

The release of the active compound from the matrix was studied and results encourage the use of the material as a potential candidate in active food packaging.

Some metals or metal-oxides show antimicrobial properties that several researchers have exploited for food applications. The amount of release of metal particles is the real challenge to meet the legislation of health institutions (EFSA, FDA).

Silver based nanocomposites of poly (3-hydroxybutyrate-co-3-hydroxyvalerate) (PHBV) and silver nanoparticles was studied [102] showing antimicrobial properties against pathogens such as *S. enterica* and *L. monocytogenes* and oxygen permeability in respect to the native polymer. PHBV was used also in composite with ZnO at various particle dimensions [103,104,105].

A recent work [106] reports an innovative process for deposition of copper-containing hybrid organic-inorganic thin film that improves the antimicrobial activity of the copper at the level of silver.

Some materials for active packaging have already been used in the food industry to preserve highly perishable foods, especially for meat, poultry, seafood, and their derivatives [107,108,109] but no application has been reported for foods prepared with cook–chill technology.

These packaging materials include substances used against microbial growth, oxidation processes or contain oxygen scavengers, carbon dioxide emitters and absorbers, moisture regulators, flavour releasers and absorbers.

## 6. Conclusions

Food safety became a topic in scientific publications because of the growing interest of the whole society on nutrition themes and rational exploitation of food resources. By providing an analysis of the literature on food technology we focused our attention on innovative packaging technology and food bio-preservatives aimed to increase the shelf-life of cooked chill foods.

Despite the developments and the strong correlation between the sector of cook–chill procedures and food active packaging research, at the present time there are no reported examples of foods cooked with cook–chill technology and stored in food active packaging. These emerged as new fields of investigation that may lead to commercially viable products to improve sustainable and healthy food system production taking advantage of both manufacturers and consumers.

Since we are certain of the strong correlation between the two sectors, this paper could be a starting point for future investigations aimed to focus light on scientific findings of innovative food technology to raise awareness among food industry managers and stakeholders to the use of advanced food technology able to produce more sustainable and healthy food systems.
